# Generalized Hypotonia Revealing Spinal Muscular Atrophy Type 2: The First Case Reported From the Dominican Republic and a Review of the Literature

**DOI:** 10.7759/cureus.11464

**Published:** 2020-11-12

**Authors:** Rubén Blanco, Jessie Pichardo, Hassan Abdullah

**Affiliations:** 1 Neurology, Pontificia Universidad Católica Madre y Maestra, Santiago, DOM; 2 Medicine, Pontificia Universidad Católica Madre y Maestra, Santiago, DOM; 3 Neurology, University of Alabama, Birmingham, USA; 4 Medicine, Nishtar Medical University, Multan, PAK

**Keywords:** hypotonia, electroneuromyography, motor neuron, muscular atrophy, survival motor neuron gene

## Abstract

Spinal muscular atrophy (SMA) is a rare, inherited autosomal recessive disease. Histopathological shreds of evidence related to the condition have suggested degenerative changes at the level of the spinal cord and brain stem. Deletions or mutations in the survival motor neuron 1 (SMN1) gene are the underlying cause of this disease. It is characterized by hypotonia, muscular atrophy, areflexia, fasciculations, and flaccid paralysis. It is further classified into five variants, depending upon the patient's age and clinical features.

In this report, we present a rare case of SMA type 2 in a one-year-old female infant who presented with generalized hypotonia and axial body weakness. Besides clinical evaluation, her genetic analysis confirmed that she had a deletion of one of the SMN1 genes. Hence, the diagnosis of SMA type 2 was confirmed.

Our study aims to emphasize that clinicians must consider this rare entity whenever a patient presents with the signs and symptoms mentioned above. As the most common cause of death in this disease is respiratory depression, an early diagnosis would prevent complications and help in the parents' genetic counseling.

## Introduction

Spinal muscular atrophy (SMA) is an inherited autosomal recessive neuromuscular disorder characterized by degeneration of anterior horn cells in the spinal cord and brainstem motor nuclei. In 95% of the cases, it is caused by deletions or mutations in the survival motor neuron 1 (SMN1) gene, resulting in little to no function in the SMN protein, which is critical for the maintenance of motor neurons [1]. The loss of the SMN1 protein is partially compensated for by SMN2 protein synthesis [2]. Disease severity in SMA generally correlates inversely with SMN2 copy number, which varies from 0 to 8 in the normal population, and to a lesser degree with the level of SMN protein [3]. The incidence of SMA ranges from 4-10 per 100,000 live births [4].

SMA is the most common monogenic cause of infant mortality [5]. It results in severe, symmetric, progressive muscular atrophy of the lower motor neuron type, and limb and bulbar muscle weakness. Patients with SMA are classified into five subtypes (type 0, 1, 2, 3, and 4) according to the age of onset and degree of severity. In this report, we present a case of SMA type 2 in a one-year-old child. To the best of our knowledge, this is the first case of its kind reported from the Dominican Republic. We present a detailed clinical and neurophysiological evaluation of this hypotonic infant.

## Case presentation

**Figure 1:**
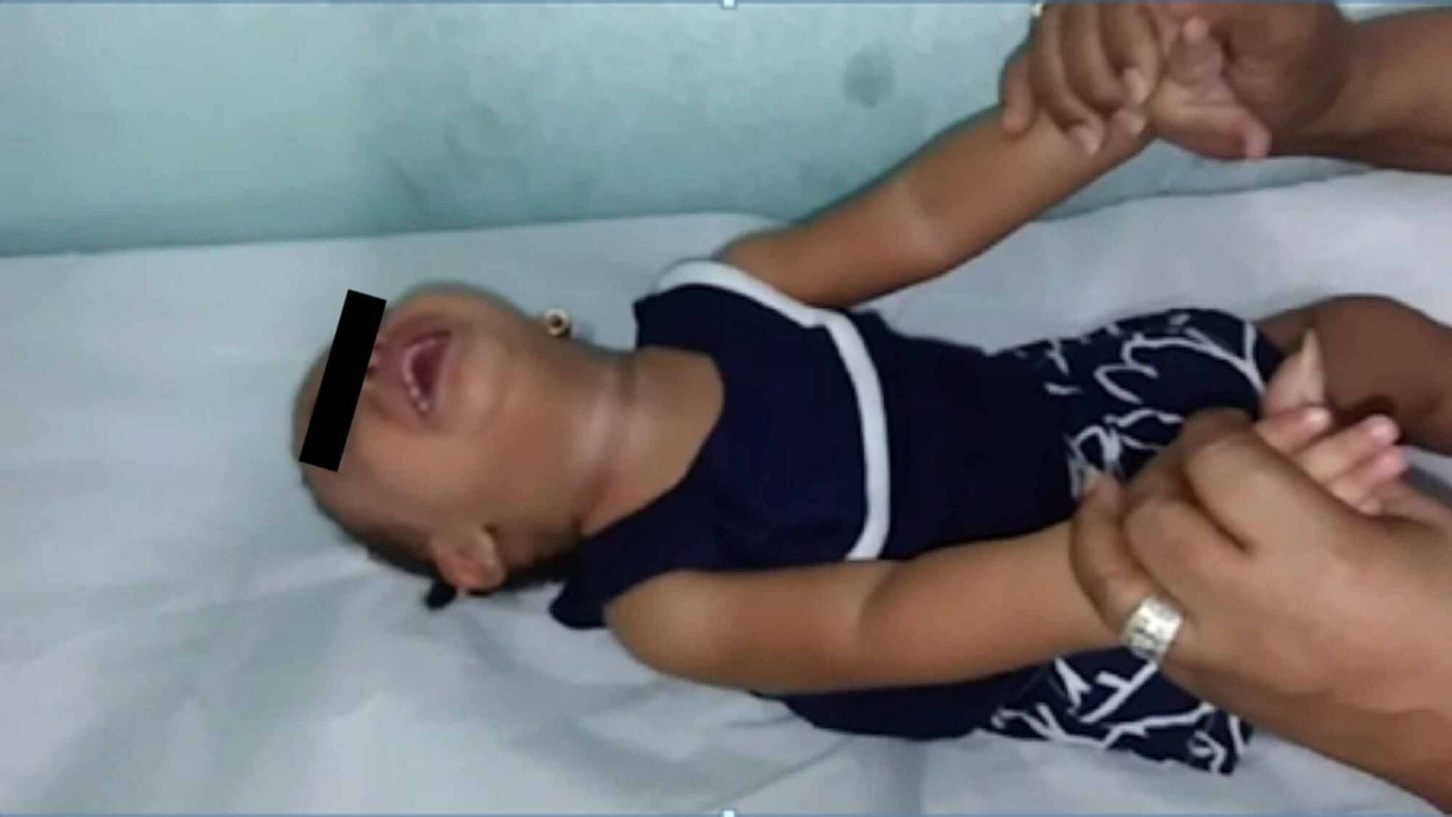
Poor head and neck control

A one-year-old girl was brought to our hospital with generalized weakness and difficulty in holding her neck. Her pregnancy and birth history were unremarkable. She had no family history of neuromuscular disorders, neurologic disorders, or congenital malformations. At the time of admission, she was vitally stable, and her general physical examination was unremarkable. The neurological assessment revealed absent deep tendon reflexes and poor head and neck control (Figure 1).

**Figure 2:**
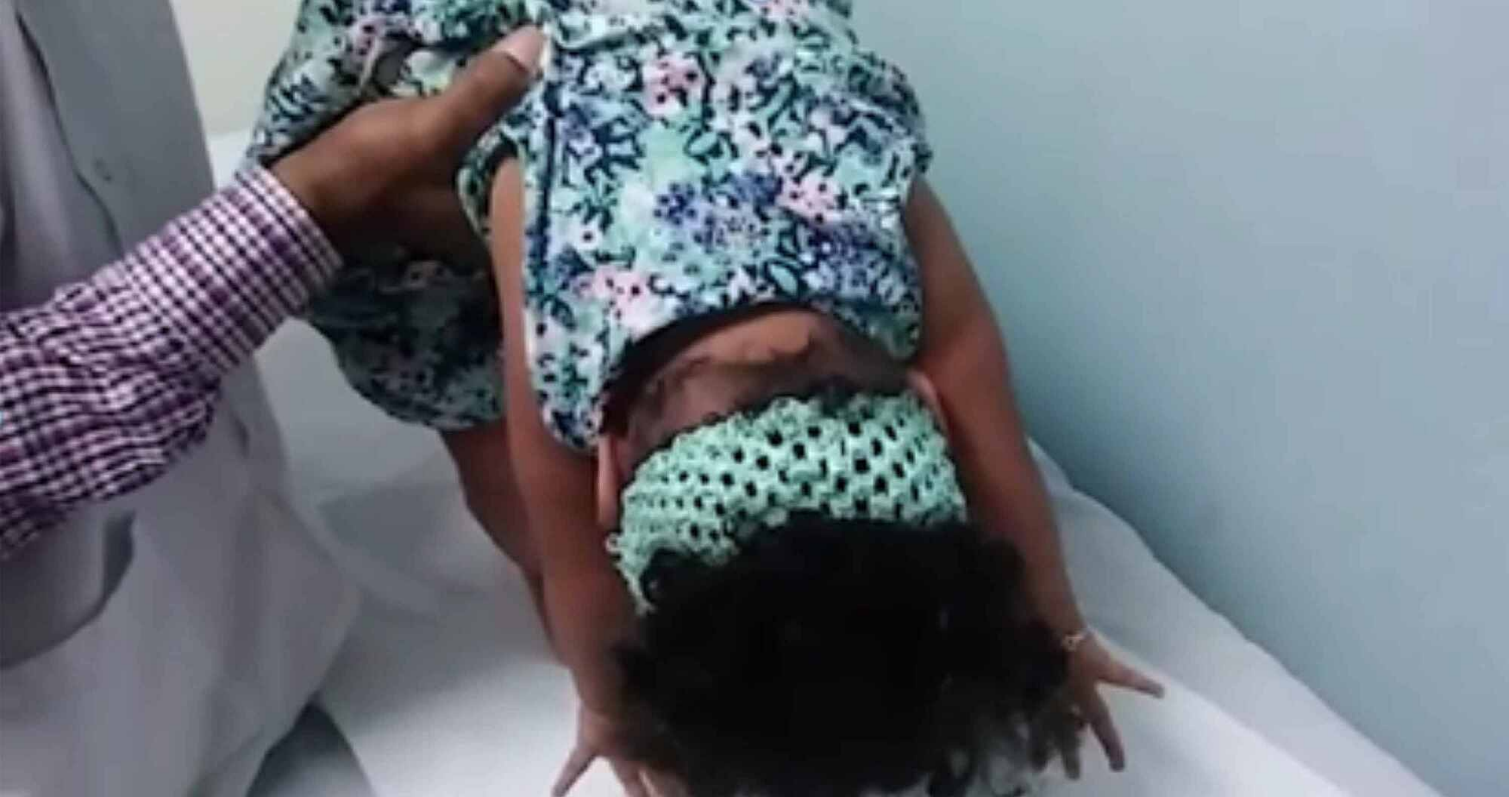
Generalized hypotonia and complete axial weakness

She had generalized hypotonia and complete axial weakness (Figure 2). She also had limb weakness predominantly involving lower limbs and a weak cry.

**Figure 3:**
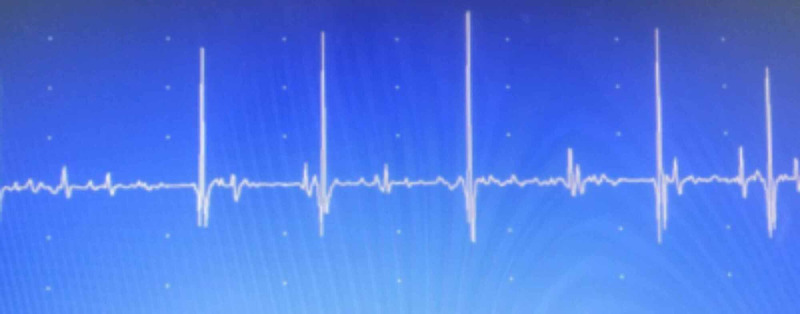
Neurogenic signs of atrophy in the deltoid muscle

**Figure 4:**
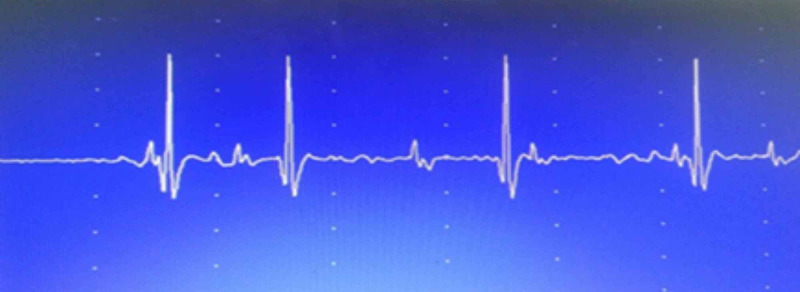
Neurogenic signs of atrophy in the tibialis anterior muscle

Laboratory analyses revealed normal complete blood counts (CBC), liver and kidney function tests, serum electrolytes, and creatine kinase (CK) levels. Electroneuromyography (ENMG) is a technique that is used to detect, localize, and define nerve and muscle disorders. It has two methods, i.e., electroneurography (ENG) and electromyography (EMG). A four extremities ENG was performed and showed reduced compound muscle action potential (CMAP) in all nerves. The needle EMG exam demonstrated the absence of spontaneous activity. The recruitment pattern was discrete or reduced, and polyphasic changes were found in all muscles. Figures 3, 4 show the ENMG findings in the deltoid and tibialis anterior muscle respectively.

Based on the history and clinical examination findings, SMA was our provisional diagnosis at that time. For confirmation, we recommended a genetic analysis for the patient. An SMN1 copy number analysis (genetic test) showed a C.835-2A>G pathogenic variant on her only copy of SMN1 (Table 1). She had two copies of the survival motor neuron 2 (SMN2) gene. These results were consistent with the diagnosis of SMA.

**Table 1:** SMN1 copy number analysis SMN1: survival motor neuron 1

Genetic test	SMN1 copy number analysis
Sample type	Peripheral blood
Results	C.835-2A>G pathogenic variant on her only copy of SMN1

As far as the outcome of our case is concerned, unfortunately, our patient developed severe respiratory depression and died afterward.

## Discussion

SMA is associated with varying degrees of presentation. It is a spectrum of disorders with overlapping symptoms characterized by degenerative changes at the spinal cord and brain stem levels. It can be associated with SMN1 gene mutations. SMN1-associated SMA is classified into five types based on the age of presentation and degree of severity. Some other variants are not associated with the SMN1 gene mutation. These non-5q SMAs include nonproximal SMA, bulbar palsy, spinobulbar muscular atrophy, and infantile SMA variants.

In Table 2, we summarize some of the studies published on the topic of SMA. After a thorough review of the literature, we found some case reports that deal specifically with SMA type 2.

**Table 2:** Literature review related to spinal muscular atrophy UKN: unknown; SMA: spinal muscular atrophy

Author	Year	SMA type and inheritance	Associated findings	Outcome
Oates et al. [6]	2012	SMA type 1 with the autosomal dominant inheritance pattern	Congenital developmental dysplasia of the right hip	Died at the age of 14 months
Zheng and Lin [7]	2013	UKN	Open bite	UKN
Massucato et al. [8]	2015	SMA type 2, autosomal recessive inheritance	Respiratory pathologies and crippling muscular atrophy	UKN
Savaş et al. [9]	2015	Congenital SMA with predominant upper limb involvement, UKN inheritance pattern	Congenital contractures of both the upper and lower limbs	Stabilized
Reid et al. [10]	2016	SMA type 1, UKN inheritance pattern	Respiratory arrest	Did not survive
Fleming et al. [11]	2016	Autosomal dominant congenital SMA	Right talipes equinovarus	Stabilized
Koul et al. [12]	2017	SMA type 4 with the autosomal recessive inheritance pattern	Bilateral pes cavus	Stabilized
Shervin Badv et al. [13]	2019	SMA progressive myoclonic epilepsy subtype (SMA-PME), UKN inheritance	Progressive myoclonic epilepsy	Stabilized
Cooper et al. [14]	2019	SMA type 3, autosomal recessive inheritance	Scoliotic deformity	Stabilized
Cooper et al. [14]	2019	SMA type 4, autosomal recessive inheritance	High-arched feet and hammertoes	Stabilized
Mayer and Campbell [15]	2019	SMA type 2, autosomal recessive inheritance	Bilateral superior rib cage decline	Stabilized

Massucato et al. have reported a case of a three-year-old female child who was diagnosed with SMA type 2 when she was just six months old [8]. As per her parents, she had difficulty keeping seated with generalized hypotonia and had respiratory pathologies similar to ones in our patient. Contrastingly, she developed severe muscular atrophy.

A case study published online in Orthopaedics Update has described SMA type 2 in a four-year-old male child [15]. Unlike our case, he had an evident musculoskeletal deformity, bilateral superior rib cage decline with scoliosis, which was compromising his respiratory function.

The presenting complaints may overlap between different subtypes of SMA. But the age of onset and the severity of symptoms can help differentiate between subtypes. Reid et al. have reported a case of SMA type 1 where the patient presented with the same set of symptoms as in our case, but the distinguishing feature of that case was the age of onset. Like our case, the patient went under respiratory depression and died [10].

The inheritance pattern of SMN-associated SMAs is autosomal recessive. However, Oates et al. have reported a case of autosomal dominant congenital SMA. It had almost the same set of symptoms, but the inheritance pattern was different. It was thought to be caused by mutations in TRPV4 genes. Unfortunately, the child did not survive and died at the age of 14 months [6].

## Conclusions

Based on our findings on SMA, clinical and electrophysiological evaluations remain the best form of initial assessment in guiding the physicians to an accurate diagnosis and their choice of related genetic analysis. We hope that this review will enlighten clinicians in our country about this condition and promote the use of ENMG studies in suspected patients.
